# Comparison of neuroimaging features of histiocytic neoplasms with central nervous system involvement: a retrospective study of 121 adult patients

**DOI:** 10.1007/s00330-023-09724-8

**Published:** 2023-05-16

**Authors:** Xiaoyuan Fan, Ting Liu, Zhiwen Zhang, Jian Sun, Na Niu, Chenhui Mao, Fengdan Wang, Jian Li, Daobin Zhou, Xinxin Cao, Zhengyu Jin, Feng Feng

**Affiliations:** 1 Department of Radiology, Peking Union Medical College Hospital, Chinese Academy of Medical Sciences and Peking Union Medical College, Beijing, China; 2 Department of Hematology, Peking Union Medical College Hospital, Chinese Academy of Medical Sciences and Peking Union Medical College, Beijing, China; 3 Department of Pathology, Peking Union Medical College Hospital, Chinese Academy of Medical Sciences and Peking Union Medical College, Beijing, China; 4 Department of Nuclear Medicine, Peking Union Medical College Hospital, Chinese Academy of Medical Sciences and Peking Union Medical College, Beijing, China; 5 Department of Neurology, Peking Union Medical College Hospital, Chinese Academy of Medical Sciences and Peking Union Medical College, Beijing, China; 6 State Key Laboratory of Difficult, Severe and Rare Diseases, Peking Union Medical College Hospital, Chinese Academy of Medical Sciences and Peking Union Medical College, Beijing, China

**Keywords:** Central nervous system, Erdheim-Chester disease, Langerhans cell histiocytosis, Rosai-Dorfman disease, Magnetic resonance imaging

## Abstract

**Objectives:**

To compare neuroimaging characteristics of three types of histiocytoses, namely Langerhans cell histiocytosis (LCH), Erdheim-Chester disease (ECD), and Rosai-Dorfman disease (RDD), with central nervous system (CNS) involvement.

**Methods:**

A total of 121 adult patients with histiocytoses (77 LCH, 37 ECD, and 7 RDD) and CNS involvement were retrospectively included. Histiocytoses were diagnosed based on histopathological findings combined with suggestive clinical and imaging features. Brain and dedicated pituitary MRIs were systematically analyzed for tumorous, vascular, degenerative lesions, sinus, and orbital involvement and for hypothalamic pituitary axis involvement.

**Results:**

Endocrine disorders, including diabetes insipidus and central hypogonadism, were more common in LCH patients than in ECD and RDD patients (*p* < 0.001). In LCH, tumorous lesions were mostly solitary (85.7%), located in the hypothalamic pituitary region (92.9%), and without peritumoral edema (92.9%), while in ECD and RDD, tumorous lesions were often multiple (ECD: 81.3%, RDD: 85.7%), their distribution was more widespread with meninges mostly involved (ECD: 75%, RDD: 71.4%), and they most likely presented with peritumoral edema (ECD: 50%, RDD: 57.1%; all *p* ≤ 0.020). Vascular involvement was an exclusive imaging characteristic of ECD (17.2%), which was not observed in LCH or RDD; this was also associated with a higher risk of death (*p* = 0.013, hazard ratio = 11.09).

**Conclusion:**

The typical characteristic of adult CNS-LCH was endocrine disorders with radiological findings limited to the hypothalamic pituitary axis. The pattern of multiple tumorous lesions with predominant involvement of meninges was the main manifestation of CNS-ECD and CNS-RDD, while vascular involvement was pathognomonic for ECD and associated with poor prognosis.

**Clinical relevance statement:**

Involvement of the hypothalamic-pituitary axis is the typical imaging characteristic of Langerhans cell histiocytosis. Multiple tumorous lesions, predominantly involving but not limited to meninges, occur in most Erdheim-Chester disease and Rosai-Dorfman disease patients. Vascular involvement occurs only in Erdheim-Chester disease patients.

**Key Points:**

*• The different distribution patterns of brain tumorous lesions can help differentiate among LCH, ECD, and RDD.*

*• Vascular involvement was an exclusive imaging finding of ECD and was associated with high mortality.*

*• Some cases with atypical imaging manifestations were reported to further expand the knowledge on these diseases.*

**Supplementary Information:**

The online version contains supplementary material available at 10.1007/s00330-023-09724-8.

## Introduction

Histiocytoses, primarily including Langerhans cell histiocytosis (LCH), Erdheim-Chester disease (ECD), and Rosai-Dorfman disease (RDD), are rare diseases characterized by the accumulation of mononuclear phagocytic cells (dendritic cells and macrophages) in various tissues and organs [1] such as the bones, skin, central nervous system (CNS), and cardiovascular and respiratory systems, among which CNS involvement is associated with increased disability and mortality, especially in patients with ECD [2, 3]. However, the misdiagnosis rate of CNS histiocytic disorders is quite high because of their complexity [4, 5].

The rarity of CNS histiocytoses and their varying clinical presentations make the diagnosis of these diseases extremely challenging. The clinical spectrum ranges from acute or rapidly progressive symptoms (e.g., stroke or seizures) to a chronic neurodegenerative process [4]. The difficulty of differential diagnosis among the different types of histiocytoses can also lead to a delayed diagnosis. Familiarity with the clinical and imaging features of CNS involvement in histiocytic neoplasms is helpful for diagnosis, differential diagnosis, treatment monitoring, and prognosis prediction. Thus, it is important to recognize the radiological characteristics of CNS involvement in different types of histiocytoses.

The histopathological appearance of histiocytic disorders is frequently non-specific, given that the neoplastic histiocytic cells are often sparsely distributed and obscured by fibrosis and mixed reactive inflammation [1, 6]. Moreover, histiocytosis overlap is not uncommon [7]. The histology and phenotype of biopsy lesions can be indistinguishable in the setting of mixed histiocytosis. Even in patients with clinically highly suspected histiocytoses, it may be difficult to determine the definitive type based on pathology alone. Histiocytoses need to be diagnosed on the basis of histological biopsy in combination with appropriate clinical manifestation and radiological findings [4]. Hence, it is crucial to accurately identify and compare the clinical and radiological characteristics of CNS involvement in histiocytoses.

Previous studies [8–14] on CNS involvement in histiocytoses often focused on only one type of histiocytosis. Consequently, the similarities and differences of CNS lesions among LCH, ECD, and RDD have not been systematically analyzed. ECD is more common in adults, although the actual incidence is unknown, and thus, the differential diagnosis of different types of histiocytoses is often required for adult patients in clinical practice. Nevertheless, most literatures report only on CNS involvement in LCH or mainly included pediatric subjects because of the extremely low incidence of adult LCH. A few studies on adult LCH patients with CNS involvement have been published as case reports [11, 15, 16].

Therefore, this study aimed to summarize and compare the neuroimaging characteristics of the three types of histiocytosis, namely LCH, ECD, and RDD, in adult patients with CNS involvement. In addition, some cases with atypical imaging manifestations were also reported to further expand the knowledge on these diseases.

## Materials and methods

### Patient population

This retrospective study was approved by the medical ethics committee of our hospital, and informed consent was waived. The Histiocytosis Working Group of our center that included hematologists, radiologists, pathologists, and neurologists conducted regular multidisciplinary consultations. Consecutive patients with a diagnosis of histiocytosis in our hospital between December 2005 and September 2021 were retrospectively reviewed from the prospective database for histiocytosis. Histiocytoses were diagnosed on the basis of histological findings together with clinical and radiological characteristics [17]. Brain and/or dedicated pituitary MRI examination was performed on patients who had neurologic or endocrine symptoms and signs or abnormal laboratory test results for hormones. Adult patients (≥ 18 years old) with a diagnosis of LCH, ECD, or RDD and CNS involvement determined by MRI at any time during the course of the disease were included. Since the main purpose of this study was to summarize and differentiate the neuroimaging features of different types of histiocytosis, patients with mixed histiocytosis or normal MR images were excluded. Thus, the exclusion criteria were as follows: (1) mixed histiocytosis; (2) without brain and pituitary MR images; (3) normal MR images; and (4) younger than 18 years at symptom onset.

### Clinical data

Because of varying clinical manifestations, these patients initially visited different outpatient departments. If histiocytosis was suspected, the patients were referred to an experienced specialist (X.C.) in histiocytic disorders for thorough evaluation, treatment, and follow-up. Demographic characteristics, duration of symptoms prior to diagnosis, clinical manifestations, and involved organs were assessed. The presence of the *BRAF* V600E mutation was detected by polymerase chain reaction, immunohistochemistry, or next-generation sequencing, as previously described [18, 19] in 37 LCH patients and 32 ECD patients who were willing to receive *BRAF* inhibitor therapy. All patients were followed up every 3 to 6 months in the outpatient clinic. Outcome was defined as death from histiocytosis-related CNS involvement during follow-up.

### MRI protocol

Enhanced and unenhanced MRI of the brain and pituitary gland was performed on a 3.0 T MRI scanner (GE Discovery 750, GE Medical System) equipped with an 8-channel head coil. Brain MRI was performed in 23 LCH patients (23 enhanced brain MRI), 29 ECD patients (24 enhanced and 5 unenhanced brain MRI), and 7 RDD patients (7 enhanced brain MRI). Dedicated pituitary MRI was performed in 68 LCH patients (63 enhanced and 5 unenhanced pituitary), 21 ECD patients (20 enhanced and 1 unenhanced pituitary), and 2 RDD patients (1 enhanced and 1 unenhanced pituitary). The detailed imaging parameters are provided in Table S1.

### Image analysis

Patients who underwent brain MRI were analyzed for the following signs according to previous studies [12, 20]: (1) sinus involvement; (2) orbital involvement; (3) extra- or intracranial tumorous lesions; (4) vascular sheathing with or without stroke; and (5) degenerative pattern. Sinus and orbital involvements were defined as iso-hypointense signal on both T1- and T2-weighted images (WI) with intense enhancement [21]. The following characteristics of the tumorous lesions were recorded: number, location, size (micronodular lesions  < 3 mm in diameter, nodular lesion 3–10 mm in diameter, mass lesions  > 10 mm in diameter [21]), signal intensity, enhancement, and peritumoral edema. The degenerative pattern included cortical, midbrain, or cerebellum atrophy and hyperintense signals on T2-weighted and FLAIR images without gadolinium enhancement [12, 20]. The 0–3-point scale developed by Hsu et al [22] was adopted to assess cortical and cerebellar atrophy: grade 0, no atrophy; grade 1, mildly dilated sulci; grade 2, substantially dilated sulci and loss of volume; grade 3, evident widening of the sulci and knife blade appearance of the cortex. Grade  ≥ 2 was considered atrophy [22]. The midbrain was evaluated to be atrophied when widened interpeduncular cistern, concave tegmentum, and flat/concave superior midbrain profile were observed [23]. Reference images for each rating scale were available during the evaluation.

Patients who underwent dedicated pituitary MRI were analyzed for the following signs: (1) thickened pituitary stalk, (2) loss of the posterior pituitary bright spot, (3) pituitary atrophy, and (4) abnormal pituitary enhancement. The pituitary stalk was measured at the level of median eminence and the midpoint between the median eminence and dorsum sellae in coronal images [24]. A thickened pituitary stalk was defined when the thickness was  ≥ 4 mm at the median eminence level or  ≥ 3 mm at the midpoint level [9]. The pituitary gland was considered to show atrophy if the largest vertical dimension of the pituitary was  ≤ 4 mm [10]. Abnormal pituitary enhancement was defined as heterogeneous signal intensity of the entire pituitary parenchyma on enhanced T1WI [10]. Physiological changes in the pituitary size and Rathke’s cyst were considered to be normal.

Two radiologists (X.F. and F.W. with 5 and 15 years of experience in neuroradiology, respectively) were trained before performing image analyses and reviewed the brain and pituitary MR images independently. Then, the two radiologists reached an agreement by reviewing and discussing the discrepancies after their first independent reading. In addition, to assess intra-observer variability, one observer (X.F.) reviewed the same images 6 months apart.

### Statistical analysis

Data were analyzed using the SPSS software (version 25.0; IBM) and MedCalc Statistical Software (version 20.09; MedCalc Software). Continuous variables in the study were non-normally distributed and expressed as median and quartiles. Categorical variables were expressed as frequencies and ratios. Cohen’s kappa values were calculated for the inter-observer and intra-observer agreements of the imaging findings.

Differences between the groups were tested using the Mann-Whitney test for continuous data and Fisher’s exact test for qualitative data. It is worth mentioning that since only patients with CNS-histiocytosis were included for analysis, the frequencies reported in our study cannot represent the incidence of CNS lesions in patients with histiocytosis, but rather the incidence of lesions in patients with CNS-histiocytosis. Specifically, comparisons among groups were as follows: (1) clinical characteristics were compared in all three groups of patients (LCH: *n* = 77, ECD: *n* = 37, RDD: *n* = 7); (2) incidence of *BRAF* V600E was compared between 37 LCH patients and 32 ECD patients who have completed genetic testing; (3) incidences of sinus involvement, orbital involvement, tumorous lesions, vascular sheathing/stroke, and degenerative pattern were compared among patients who underwent brain MRI (LCH: *n* = 23, ECD: *n* = 29; RDD: *n* = 7); (4) incidences of thickened pituitary stalk, loss of the posterior pituitary bright spot, and pituitary atrophy were compared among patients who underwent pituitary MRI (LCH: *n* = 68, ECD: *n* = 21, RDD: *n* = 2), while incidence of abnormal pituitary enhancement was compared only among patients with enhanced pituitary MRI (LCH: *n* = 63, ECD: *n* = 20, RDD: *n* = 1); (5) further comparisons of characteristics of tumorous lesions were performed among 37 patients (LCH: *n* = 14, ECD: *n* = 16, RDD: *n* = 7) with CNS tumors on brain MRI. Specific neurodegeneration patterns were compared between 11 LCH patients and 18 ECD patients. Because degenerative changes were observed only in one RDD patient in a novel form, it was reported in detail but not included for the comparison; (6) extra-CNS involvements were compared in all included patients.

The Kaplan-Meier analysis is recommended for survival analysis irrespective of sample size; thus, it is particularly suited to rare diseases [25]. To evaluate the association between neuroimaging markers and clinical outcome, the Kaplan-Meier analysis and log-rank test were used to compare the survival curves [26]. All tests were two-sided, and *p* < 0.05 was considered to be statistically significant; Bonferroni’s correction was used for multiple comparisons.

## Results

### Study population and clinical characteristics

**Fig. 1:**
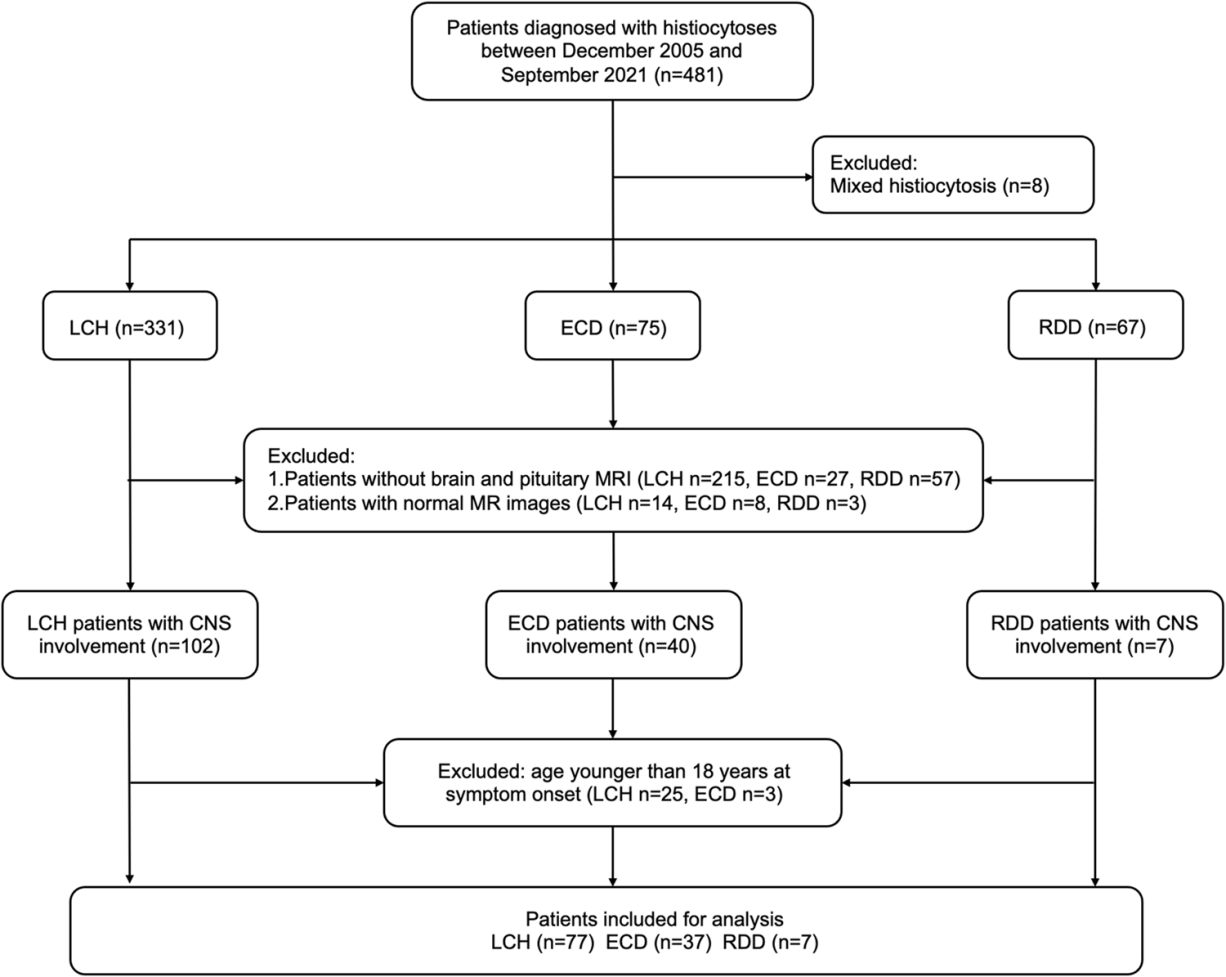
Flowchart of patient selection

A total of 121 adult patients with CNS involvement, including 77 LCH patients, 37 ECD patients, and 7 RDD patients, were finally included (Fig. 1). Their clinical characteristics are shown in Table 1. LCH patients were younger than ECD patients, both at symptom onset (28 [22, 36] years vs. 44 [33.5, 49.5] years, *p* < 0.001) and at diagnosis (30 [25, 41] years vs. 46 [38, 53] years, *p* < 0.001). Endocrine disorders, including diabetes insipidus (LCH vs. ECD: 89.6% [69/77] vs. 37.8% [14/37], *p* < 0.001; LCH vs. RDD: 89.6% [69/77] vs. 0, *p* = 0.078) and central hypogonadism (LCH vs. ECD: 51.9% [40/77] vs. 5.4% [2/37], *p* < 0.001; LCH vs. RDD: 51.9% [40/77] vs. 0, *p* = 0.013), were more common in patients with LCH than in ECD and RDD patients. The incidence of headache was higher in ECD (18.9%, 7/37) than that in LCH (2.6%, 2/77, *p* = 0.005). The *BRAF* V600E mutation was more common in ECD (68.8%, 22/32) with CNS involvement than in LCH (27.0%, 10/37, *p* = 0.001). Involvement of other organs is shown in Table 1 and detailed in Appendix in the supplementary material.

**Table 1:** Clinical and neuroimaging characteristics of patients with CNS histiocytoses

	LCH (*n* = 77)	ECD (*n* = 37)	RDD (*n* = 7)	*p* value
Gender, male	48 (62.3)	18 (48.6)	6 (85.7)	0.143
Age at symptom onset, years*	28 [22, 36]	44 [33.5, 49.5]	36 [30, 60]	< 0.001
Age at diagnosis, years*	30 [25, 41]	46 [38, 53]	37 [33, 61]	< 0.001
Time to diagnosis, months*	18.8 [8.2, 51]	44.1 [15.8, 102.4]	41 [12.5, 53.8]	0.028
Neurological symptoms				
Diabetes insipidus*	69 (89.6)	14 (37.8)	0	< 0.001
Central hypogonadism*	40 (51.9)	2 (5.4)	0	< 0.001
Vision loss, visual field defect	4 (5.2)	3 (8.1)	2 (28.6)	0.098
Ataxia	1 (1.3)	2 (5.4)	1 (14.3)	0.073
Headache*	2 (2.6)	7 (18.9)	1 (14.3)	0.016
Neurological deficits	0	3 (8.1)	1 (14.3)	0.031
Seizures	0	1 (2.7)	1 (14.3)	0.039
Pseudobulbar palsy	1 (1.3)	2 (5.4)	1 (14.3)	0.073
*BRAF* V600E, number	37	32	NA	
Positive*	10 (27.0)	22 (68.8)	NA	0.001
Brain MRI, number	23	29	7	
Sinus involvement	2 (8.7)	10 (34.5)	1 (14.3)	0.078
Orbital involvement*	1 (4.3)	11 (37.9)	1 (14.3)	0.011
Tumorous lesions	14 (60.9)	16 (55.2)	7 (100)	0.083
Vascular sheathing, stroke	0	5 (17.2)	0	0.063
Degenerative pattern	11 (47.8)	18 (62.1)	1 (14.3)	0.073
Pituitary MRI, number	68	21	2	
Thickened pituitary stalk	21 (30.9)	8 (38.1)	0	0.588
Loss of the posterior pituitary bright spot	62 (91.2)	19 (90.5)	1 (50)	0.104
Pituitary atrophy	36 (52.9)	9 (42.9)	2 (100)	0.300
Abnormal pituitary enhancement^a^	6/63 (9.5)	4/20 (20)	0/1 (0)	0.334
Non-CNS involvement				
Bones*	43 (55.8)	32 (86.5)	2 (28.6)	0.001
Respiratory	41 (53.2)	18 (48.6)	1 (14.3)	0.160
Dermatologic	21 (27.3)	11 (29.7)	2 (28.6)	0.943
Cardiac*	0	13 (35.1)	0	< 0.001
Arterial*	0	17 (45.9)	0	< 0.001
Retroperitoneum, including kidneys*	0	19 (51.4)	1 (14.3)	< 0.001
Lymph nodes	25 (32.5)	6 (16.2)	3 (42.9)	0.125
Liver or spleen*	25 (32.5)	1 (2.7)	1 (14.3)	0.002

*CNS*, central nervous system; *LCH*, Langerhans cell histiocytosis; *ECD*, Erdheim-Chester disease; *RDD*, Rosai-Dorfman disease. Continuous variables were expressed as median and quartiles. Categorical variables were expressed as frequencies and ratios

^*^Statistically significant (*p* < 0.017 after Bonferroni’s correction)

^a^The neuroimaging marker is expressed as the number of positive cases/patients with enhanced pituitary MRI

### Neuroimaging features

#### Sinus and orbital involvement

Most of the inter-observer and intra-observer agreements of the described neuroimaging findings were good or excellent (Appendix Table S2). Neuroimaging characteristics of the three types of histiocytoses are shown in Table 1. Sinus (34.5%, 10/29) and orbital (37.9%, 11/29) involvements were relatively common in ECD patients. The frequency of orbital involvement was higher in ECD (37.9%, 11/29) than that in LCH (4.3%, 1/23, *p* = 0.002).

#### Tumorous lesions

**Fig. 2:**
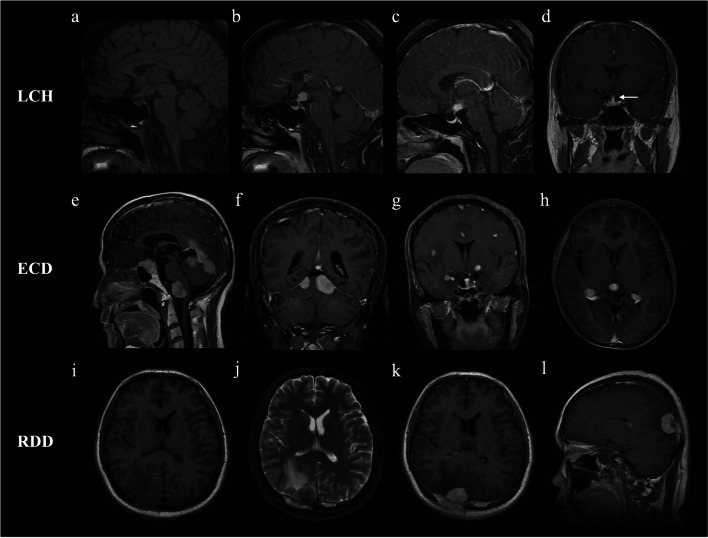
Representative cases of LCH, ECD, and RDD with tumorous lesions in the central nervous system. **a**, **b** A 41-year-old man with diabetes insipidus. T1-weighted image (WI) shows the loss of the posterior pituitary bright spot (**a**), with a single mass in the hypothalamus and infundibulum on enhanced T1WI **b**. **c** Enhanced T1WI of a 21-year-old woman with diabetes insipidus and hypogonadism shows the hypothalamic mass and pituitary atrophy. **d** Thickened pituitary stalk (arrow) was observed in a 21-year-old man with diabetes insipidus. **e**–**f** Enhanced T1WIs of a 50-year-old man with diabetes insipidus show multiple mass lesions in the saddle area and meninges. **g**–**h** Enhanced T1WIs of a 49-year-old woman show multiple tumorous lesions in the hypothalamus, infundibulum, and parenchyma with peritumoral edema. Tumorous lesions also appeared in the pineal gland and choroid plexus. **i**–**l** Classic imaging manifestations in a 33-year-old RDD patient include meningioma-like mass lesions in the bilateral occipital lobes, peritumoral edema, marked enhancement, and dural tail sign

Among patients with abnormal brain MRI findings, tumorous lesions were observed in 60.9% (14/23) LCH patients, 55.2% (16/29) ECD patients, and all (7/7) RDD patients, and the incidence was not significantly different among the three groups (*p* = 0.083). Tumor characteristics were then compared among LCH, ECD, and RDD patients with tumorous lesions (Table 2), and we found that their distribution patterns were distinctive. The involvement of the hypothalamic pituitary axis was more common in LCH (92.9%, 13/14) than in ECD (31.3%, 5/16, *p* = 0.001) and RDD (28.6%, 2/7, *p* = 0.006) (Fig. 2a–d). However, the distribution of tumorous lesions in ECD and RDD was more widespread (Fig. 2e–l). Tumorous lesions were more commonly seen in meninges in ECD (LCH vs. ECD: 14.3% [2/14] vs. 75.0% [12/16],* p* = 0.001) and RDD (LCH vs. RDD: 14.3% [2/14] vs. 71.4% [5/7], *p* = 0.017). Tumorous lesions of the supratentorial parenchyma, paraventricular regions, choroid plexus, brainstem, and cerebellum were also observed in ECD and RDD.

**Table 2:** Characteristics of tumorous lesions in patients with CNS histiocytosis

	LCH (*n* = 14)	ECD (*n* = 16)	RDD (*n* = 7)	*p* value
Location				
Supratentorial parenchyma	1 (7.1)	6 (37.5)	2 (28.6)	0.188
Meninges*	2 (14.3)	12 (75)	5 (71.4)	0.001
Paraventricular regions*	13 (92.9)	5 (31.3)	2 (28.6)	0.001
Hypothalamic pituitary axis*	13 (92.9)	5 (31.3)	2 (28.6)	0.001
Pineal gland	0	0	1 (14.3)	0.189
Choroid plexus	0	1 (6.3)	2 (28.6)	0.132
Cerebellum	1 (7.1)	2 (12.5)	1 (14.3)	1.0
Brainstem	1 (7.1)	5 (31.3)	1 (14.3)	0.303
Number*				< 0.001
Single lesion	12 (85.7)	3 (18.8)	1 (14.3)	
Multiple lesions	2 (14.3)	13 (81.3)	6 (85.7)	
Size				
Micronodular lesion	0	3 (18.8)	1 (14.3)	0.384
Nodular lesion	5 (35.7)	6 (37.5)	2 (28.6)	1.0
Mass lesion	10 (71.4)	14 (87.5)	6 (85.7)	0.556
Peritumoral edema*	1 (7.1)	8 (50)	4 (57.1)	0.020
Enhancement pattern, number	14	14	7	
Non-/mild enhancement	0	1 (7.1)	1 (14.3)	0.671
Marked homogeneous enhancement	14 (100)	12 (85.7)	6 (85.7)	0.401
Ring/septum enhancement	0	2 (14.3)	1 (14.3)	0.401

*CNS*, central nervous system; *LCH*, Langerhans cell histiocytosis; *ECD*, Erdheim-Chester disease; *RDD*, Rosai-Dorfman disease. Micronodular lesion indicates  < 3 mm in diameter. Nodular lesion indicates 3–10 mm in diameter. Mass lesion indicates  > 10 mm in diameter

^*^Statistically significant (*p* < 0.017 after Bonferroni’s correction)

The number of tumors was also significantly different: most LCH patients (85.7%, 12/14) had single tumorous lesion, whereas ECD and RDD patients often had multiple tumorous lesions (81.3% [13/16] for ECD, *p* < 0.001; 85.7% [6/7] for RDD, *p* = 0.003). Moreover, the incidence of peritumoral edema was lower in LCH (7.1%, 1/14) than in ECD (50.0%, 8/16, *p* = 0.017) and RDD (57.1%, 4/7, *p* = 0.025). No significant difference was observed in signal characteristics, tumor size, and enhancement modes among the three groups.

#### Vascular involvement

**Fig. 3:**
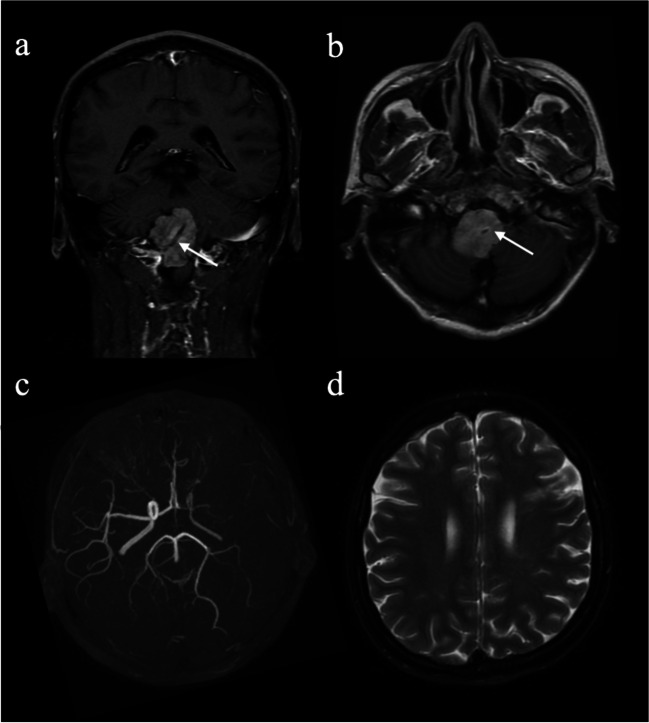
Vascular involvement in ECD patients. **a**, **b** Enhanced T1-weighted images show tumoral infiltration around the right vertebral artery (arrows) and compression of the medulla oblongata in a 43-year-old ECD patient. **c**, **d** In a 45-year-old man diagnosed to have ECD, MR angiography and T2-weighted image show the vaguely seen left carotid artery and complete occlusion of the left middle cerebral artery and encephalomalacia in the left frontal cortex

Vascular involvement was observed in five (17.2%) of 29 ECD patients, but not found in LCH and RDD patients. Of the five ECD patients with vascular involvement, two patients had right vertebral artery sheathing that led to compression of the medulla oblongata, and three had stenosis or occlusion of large intracranial vessels and ischemic stroke (Fig. 3).

#### Degenerative pattern

Neurodegenerative changes were observed in 11 (47.8%, 11/23) patients with LCH and 18 (62.1%, 18/29) patients with ECD. T2 hyperintensities, which were widespread from the supratentorial to infratentorial parenchyma in ECD patients, appeared only in the posterior fossa in 4 (36.4%, 4/11) LCH patients. However, LCH patients had a higher rate of cortical atrophy than ECD patients (LCH vs. ECD: 90.9% [10/11] vs. 44.4% [8/18], *p* = 0.019; Table 3).

**Table 3:** Comparison of neurodegenerative changes between Langerhans cell histiocytosis and Erdheim-Chester disease

	LCH (*n* = 11)	ECD (*n* = 18)	*p* value
T2 hyperintense signals	4 (36.4)	10 (55.6)	0.45
Cerebral white matter	0	5 (27.8)	0.126
Cerebellum	2 (18.2)	5 (27.8)	0.677
Brainstem	4 (36.4)	7 (38.9)	1.0
Atrophy	11 (100)	14 (77.8)	0.143
Cortical*	10 (90.9)	8 (44.4)	0.019
Midbrain	4 (36.4)	4 (22.2)	0.671
Cerebellum	9 (81.8)	14 (77.8)	1.0

*CNS*, central nervous system; *LCH*, Langerhans cell histiocytosis; *ECD*, Erdheim-Chester disease

^*^Statistically significant (*p* < 0.05)

**Fig. 4:**
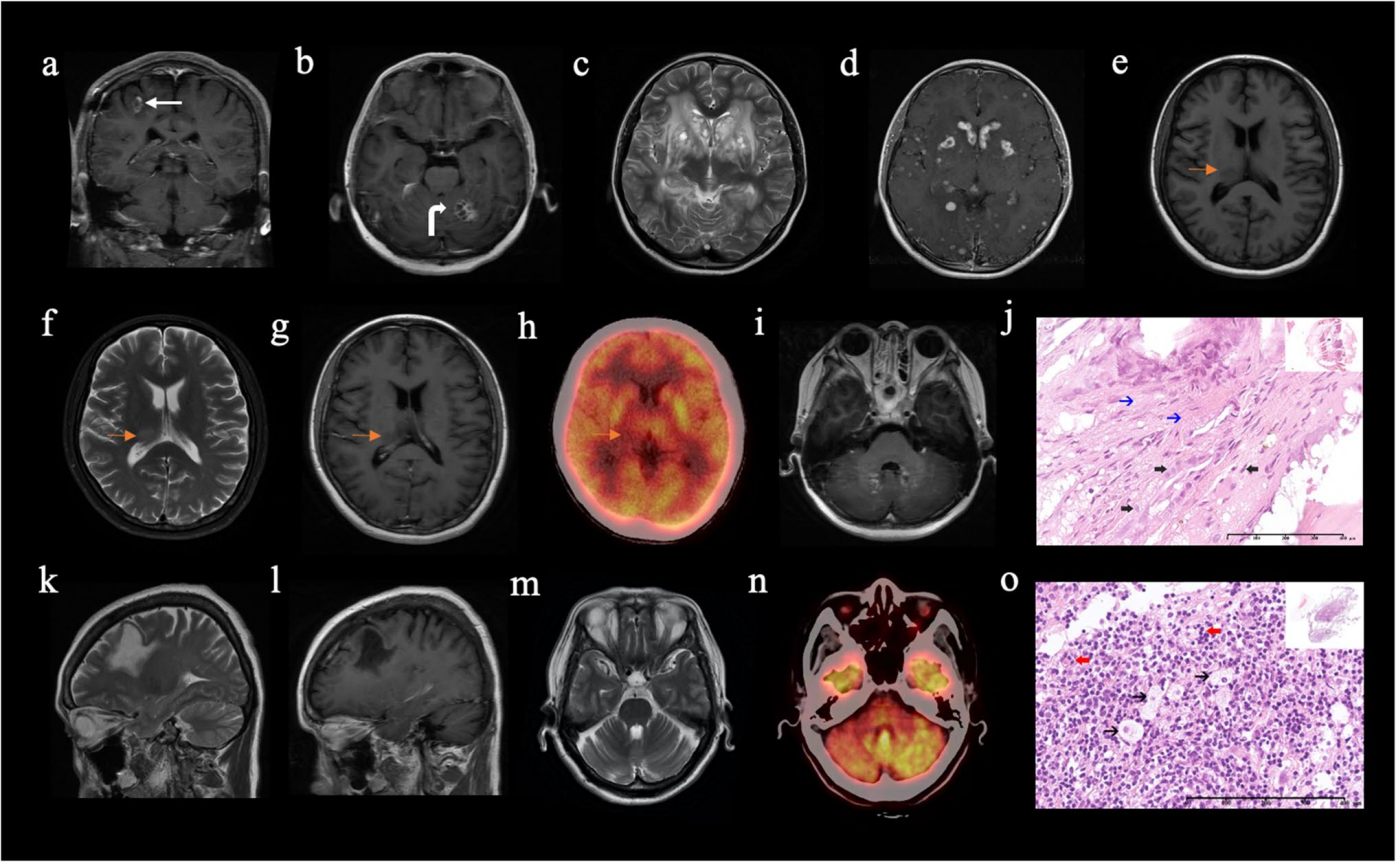
Atypical neuroimaging findings. **a** Enhanced T1-weighted image (WI) shows ring enhancements (white arrow) of the tumorous lesion in the brain parenchyma in a 47-year-old man diagnosed to have ECD. **b** Enhanced T1WI shows septum enhancement of a dura-based mass (curve white arrow) in a 53-year-old ECD patient. **c**, **d** T2 and enhanced T1WIs demonstrate multiple cystic lesions with ring enhancement in the brain parenchyma and choroid plexus in an 18-year-old patient diagnosed to have RDD. **e**–**h** In a 35-year-old female diagnosed to have ECD, T1WI (**e**), T2WI (**f**), and enhanced T1WI (**g**) images show a mass lesion (yellow arrows) in the right thalamus with slight T1-hypointensity, T2-hyperintensity, mild enhancement, and compression of the right lateral ventricle, and ^18^FDG-PET/CT (**h**) shows no radioactivity. **i**, **j** Axial enhanced T1WI (**i**) demonstrates tumorous lesions around the fourth ventricle with small, punctate gadolinium enhancements mimicking chronic lymphocytic inflammation with pontine perivascular enhancement in a 30-year-old ECD patient. Atypical neoplastic histiocytes (black thick arrows) with a single round nucleus and foamy cytoplasm can be observed in fibrous background (blue arrows) on pathological image (**j**) (hematoxylin and eosin staining;  × 400 magnification; scale bar = 400 μm). **k**–**o** In a 61-year-old female patient diagnosed to have RDD, T2WI (**k**) and enhanced T1WI (**l**) show a single large cyst without obvious enhancement in the left frontal lobe. Axial T2WI (**m**) shows cerebellar atrophy with prominent cerebellar folia and enlarged cistern. Axial ^18^FDG-PET/CT (**n**) confirms right crossed cerebellar diaschisis. On pathological image (**o**) (hematoxylin and eosin staining;  × 400 magnification; scale bar = 400 μm), histiocytes have a large vesicular nucleus and abundant clear or lightly eosinophilic cytoplasm (black thin arrows), and many of the histiocytes contain numerous intact lymphocytes or plasma cells in their cytoplasm (red arrows)

T2 hyperintensities were not observed in RDD patients, while cerebellum atrophy was detected in one RDD patient with a tumor in the left frontal lobe (Fig. 4m).

#### Hypothalamic pituitary axis involvement

In patients with hypothalamic pituitary axis involvement, the frequencies of the thickened pituitary stalk, loss of the posterior pituitary bright spot, pituitary atrophy, and abnormal pituitary enhancement were similar among the three groups without a significant difference (Table 1).

### Cases with atypical neuroimaging findings

Several cases with atypical MR imaging features were observed in ECD and RDD patients. One pattern of atypical imaging manifestation was cystic lesions with ring or septum enhancements, which were observed in both ECD and RDD patients (Fig. 4a–d). An ECD case with a single solid mass in the thalamus without any enhancement was also noted (Fig. 4e–h). Another ECD patient had tumorous lesions around the fourth ventricle with small, punctate gadolinium enhancements mimicking chronic lymphocytic inflammation with pontine perivascular enhancement responsive to steroids (CLIPPERS) (Fig. 4i–j). We also found a RDD patient who had a single large cyst without enhancement in the left frontal lobe (Fig. 4k–l). Interestingly, ^18^FDG-PET/CT showed right crossed cerebellar diaschisis, and brain MRI revealed cerebellum atrophy (Fig. 4m–o). This RDD patient could not complete right finger-nose, both hands alternate motion, and heel-knee-tibia tests.

### Association between neuroimaging features and survival outcome

**Fig. 5:**
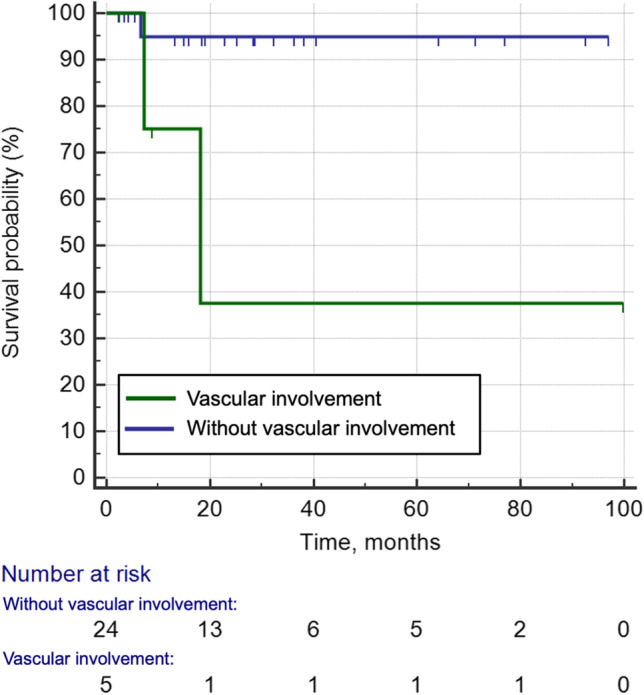
Comparison of cumulative survival rate between CNS-ECD patients with and without vascular involvement

The median duration of follow-up was 63 months (range: 1–187 months) for LCH patients, 22 months (range: 2–106 months) for ECD patients, and 24 months (range: 5–62 months) for RDD patients. Two (2.6%, 2/77) patients with LCH and 3 (8.1%, 3/37) with ECD died due to CNS disease progression. Of the 5 ECD patients with CNS vascular involvement, one died of ischemic stroke and the other died of intracerebral hemorrhage. As shown in Fig. 5, in CNS-ECD, the risk of death was higher in patients with vascular involvement than in those without (3-year survival rate: 37.5% vs. 95%, *p* = 0.013; hazard ratio: 11.09). No significant difference in the risk of death was observed between patients with and without other neuroimaging markers.

## Discussion

In the present study, we summarized and compared the neuroimaging features of three types of adult histiocytosis with CNS involvement in a retrospective, single-center series of 121 adult patients (77 patients with LCH, 37 patients with ECD, and 7 patients with RDD). Endocrine disorders and radiological findings limited to the hypothalamic pituitary axis, sometimes with neurodegenerative changes, were the typical characteristics of adult LCH patients. The pattern of multiple tumorous lesions, predominantly involving but not limited to the meninges, was the primary manifestation of CNS involvement in ECD and RDD patients, while vascular involvement was an exclusive imaging finding of ECD. We also reported several cases with atypical radiological manifestation to help clinicians raise their awareness regarding these “orphan diseases.”

The present study found that diabetes insipidus was the most common neurological symptom for both adult LCH and ECD patients with CNS involvement, but not observed in RDD patients; this finding was in line with previous studies [4]. Central diabetes insipidus can be the earliest clinical clue raising suspicion of LCH and ECD in the absence of other etiologies [3]. For the differential diagnosis, the following imaging features may be suggestive of ECD: orbital involvement, multiple tumorous lesions located beyond the hypothalamic pituitary region, peritumoral edema, vascular involvement, and supratentorial T2 hyperintensities. In contrast, a single tumorous lesion confined to the hypothalamic pituitary axis with the symptom of diabetes insipidus is the representative imaging finding of adult CNS-LCH.

Unlike LCH, ECD and RDD patients presented with various neurological symptoms. ECD patients had the highest heterogeneity in clinical symptoms and neurological findings, which was consistent with previous studies [12, 13]. ECD-specific features that were distinguishable from those of RDD included vascular sheathing, stroke, and degenerative T2 hyperintensities. Vascular involvement was an exclusive neuroimaging finding of ECD which was not observed in LCH and RDD patients. The neuroimaging feature of most CNS-RDD patients is multiple well-defined, dural-based, and extra-axial masses mimicking meningioma, with peritumoral edema and marked enhancement [9, 14]. This typical radiological finding was observed in most RDD patients in our study. Moreover, our results emphasized that RDD tumorous lesions can actually infiltrate almost every part of the CNS, including both supra- and infratentorial and intra- and extra-axial regions. When CNS involvement presents only as the tumorous pattern, the differential diagnosis between ECD and RDD needs to focus more on organs beyond the CNS [3]. Involvements of the cardiovascular system (“coated aorta”), bone, and retroperitoneum (“hairy kidney” or fibrosis) are suggestive of ECD. Liver and spleen involvement may suggest LCH.

The degenerative pattern including brain atrophy and T2-weighted hyperintense signals in both LCH and ECD patients have been reported in previous studies [12, 20]. We found that cortical atrophy was more commonly observed in LCH than in ECD. Abnormalities of the hypothalamic-pituitary axis may affect the brain structure through metabolic abnormalities, resulting in cortical atrophy [27, 28]. The high incidence of hypothalamic pituitary involvement in LCH may explain cortical atrophy in CNS-LCH. Unlike LCH and ECD, neurodegenerative lesions and syndromes in RDD have not been systematically described in literature [1]. To the best of our knowledge, only a case of RDD with T2 hyperintensities in the cerebellum and pons was reported by Candeias et al [29]. In our study, we did not find degenerative MR signals in RDD patients, but cerebellum atrophy in a RDD patient was noted, which may result from right crossed cerebellar diaschisis secondary to the tumor in the left frontal lobe, as crossed cerebellar diaschisis sometimes leads to the morphologic changes of cerebellar atrophy [30]. Our finding suggests that neurodegenerative changes might occur in the form of cerebellar atrophy in RDD.

We also described some cases with atypical neuroimaging manifestations. Our findings suggested that cystic changes with ring or septum enhancement of tumorous lesions can be observed in ECD and RDD patients. Although the majority of ECD and RDD lesions in the brain parenchyma are markedly enhanced, they could show no enhancement at all. These presentations have rarely been reported previously. We also described an ECD patient who presented with pseudo-CLIPPERS, which was similar to a case reported by Cohen Aubart et al [12].

A literature review of 66 ECD patients found cardiovascular involvement, an overlooked feature of ECD, accounted for a significant proportion of the deaths associated with ECD [31], while such data is lacking in ECD patients with CNS involvement. Our study showed that ECD patients with cerebrovascular involvement had a higher risk of death than uninvolved ECD patients, and thus assessment of cerebral blood vessels might be recommended in CNS-ECD. However, the sample size of 5 patients with vascular involvement was small, the follow-up duration was relatively short, and sufficient clinical outcomes cannot be observed. Our results need to be verified by future studies with a larger sample size and longer follow-up duration.

Our study has several limitations. The main limitation was the retrospective nature of the study. Brain imaging and neurological evaluation were performed only in patients with related clinical manifestations or laboratory results rather than in all patients with histiocytic disorders; consequently, some asymptomatic or paucisymptomatic CNS lesions may be overlooked. However, based on our data, the frequency of CNS involvement in LCH (23.3%), ECD (49.3%), and RDD (10.5%) was consistent with that reported in previous literature (20–30% for LCH, 40–70% for ECD, and 10% for RDD) [3]. In addition, the imaging protocols were heterogeneous. Pituitary MRI was more frequently performed in LCH patients, while brain MRI was more frequently performed in ECD and RDD patients. A small number of patients were scanned without enhanced T1WI. Therefore, the neuroimaging characteristics presented in the present study may be somewhat impacted by selection bias.

Furthermore, the sample size of CNS-RDD was small because of the extremely low incidence, which may be a confounder for statistical analysis. Our results on the relationship between neuroimaging findings and prognosis also need to be verified by future studies with a larger sample size and longer follow-up duration. Finally, not all CNS lesions evaluated in the study were biopsied; however, in the process of diagnosis, medical history and various imaging findings, including ^18^FDG-PET/CT, demonstrated homology between CNS lesions and biopsied lesions.

In conclusion, distinctive neuroimaging characteristics were recognized in the three types of adult histiocytoses with CNS involvement, which will be helpful for clinicians to interpret neuroimages more comprehensively and obtain sufficient information for an accurate diagnosis of histiocytosis.

### Supplementary Information

Below is the link to the electronic supplementary material.

Supplementary file1 (PDF 52 KB)

## Abbreviations

CLIPPERS: Chronic lymphocytic inflammation with pontine perivascular enhancement responsive to steroids
CNS: Central nervous system
ECD: Erdheim-Chester disease
LCH: Langerhans cell histiocytosis
RDD: Rosai-Dorfman disease

## Declarations

### Guarantor

The scientific guarantor of this publication is Fengdan Wang.

### Statistics and biometry

We thank Lifan Zhang from Clinical Epidemiology Unit, International Epidemiology Network, Peking Union Medical College Hospital, Chinese Academy of Medical Science, for statistical advice.

### Study subjects or cohorts overlap

Some study subjects or cohorts have been previously reported in the following:

• Wang F, Cao X, Niu N et al (2019) Multisystemic imaging findings in Chinese patients with Erdheim-Chester disease. *AJR Am J Roentgenol* 213:1179-1186.

• Wang JN, Wang FD, Sun J et al (2021) Pulmonary manifestations of Erdheim-Chester disease: clinical characteristics, outcomes and comparison with Langerhans cell histiocytosis. *Br J Haematol* 194:1024-1033.

### Methodology

• retrospective

• diagnostic and prognostic study

• performed at one institution
