# Supplementary material for: Comparison of neuroimaging features of histiocytic neoplasms with central nervous system involvement: a retrospective study of 121 adult patients
Source: Eur Radiol. 2023 May 16;33(11):8031–42. doi: 10.1007/s00330-023-09724-8 (PMC10598130; doi:10.1007/s00330-023-09724-8)
Supplement: Supplementary file 1 — Supplementary file1 (PDF 52 KB) [file 330_2023_9724_MOESM1_ESM.pdf]

ELECTRONIC SUPPLEMENTARY MATERIAL

Comparison of neuroimaging features of histiocytic neoplasms with central nervous system involvement: a retrospective study of 121 adult patients

Table S1. Parameters for MRI protocols.

Protocol	Sequences	TR/TE/TI (ms)	FOV (mm)	Matrix	FA (°)	Number of slices	Slice thickness (mm)	b_{max} (s/mm^2)
**Unenhanced brain MRI**	Axial T1WI	1625/15/720	230×230	288x192	111	24	5	NA
	Axial T2WI	7394/82.4/-	230×230	320×320	142	24	5	NA
	Axial FLAIR	8000/147.2/2000	230×230	192×160	111	24	5	NA
	Axial DWI	3000/63.5/-	230×230	128×128	90	24	5	1000
	Sagittal T2WI	3978/83/-	240×216	352×256	111	24	5	NA
**Enhanced brain MRI**	Axial T1WI	1625/15/720	230×184	320×192	111	24	5	NA
	Sagittal T1WI	1625/15/720	240×216	320×192	111	24	5	NA
	Coronal T1WI	1625/15/720	220×198	288×160	111	24	6	NA
**Unenhanced pituitary MRI**	Coronal T1WI	460/9/-	190×152	320×224	111	8	3	NA
	Sagittal T1WI	460/9/-	170×170	256×192	90	24	3	NA

	Coronal T2WI	4400/89.4/-	200×200	320×320	111	20	4	NA
**Enhanced pituitary MRI**	Coronal T1WI	460/9/-	190×152	320×224	111	8	3	NA
	Sagittal T1WI	460/9/-	170×170	256×192	90	24	3	NA

Table S2. Inter-observer and intra-observer agreements of neuroimaging findings.

κ values	Brain MRI findings					Pituitary MRI findings			
	Sinus involvement	Orbital involvement	Tumorous lesions	Vascular sheathing	Degenerative pattern	Thickened pituitary stalk	Loss of the posterior pituitary bright spot	Pituitary atrophy	Abnormal pituitary enhancement
κ value 1	0.822	0.792	0.855	0.880	0.728	0.612	0.678	0.890	0.663
κ value 2	0.829	0.856	0.778	1.000	0.813	0.600	0.640	0.650	0.619

κ value 1: inter-observer agreement; κ value 2: intra-observer agreement

Table S2: (Continued)

κ values	Tumor location						Tumor number
	Supratentorial parenchyma	Meninges	Paraventricular region	Choroid plexus	Cerebellum	Brainstem	(Multiple lesions)
κ value 1	0.745	0.782	0.892	0.770	0.684	0.711	0.565
κ value 2	0.877	0.729	0.782	0.874	0.874	0.841	0.888

κ values	Tumor size			Peritumoral edema	Enhancement pattern		
	Micronodular lesion	Nodular lesion	Mass lesion		Non/mild enhancement	Marked homogeneous enhancement	Ring/septum enhancement
κ value 1	0.621	0.763	0.566	0.819	0.470	0.785	0.785
κ value 2	0.843	0.819	0.773	0.940	0.785	0.842	1.000

κ value 1: inter-observer agreement; κ value 2: intra-observer agreement

Table S2: (Continued)

κ values	T2 hyperintense signals			Atrophy		
	Cerebral white matter	Cerebellum	Brainstem	Cortical	Midbrain	Cerebellum
κ value 1	0.888	0.828	0.925	0.605	0.754	0.667
κ value 2	0.633	0.910	0.858	0.559	0.526	0.762

κ value 1: inter-observer agreement; κ value 2: intra-observer agreement.

Comparison of non-CNS findings

As shown in Table 1, cardiac involvement (13 of 37, 35.1%) and arterial (17 of 37, 45.9%) involvement were observed only in ECD patients. The involvement of bone (ECD vs. LCH: 86.5% vs. 55.8%, p=0.001; ECD vs. RDD: 86.5% vs. 28.6%, p=0.004) and retroperitoneal infiltrates (ECD vs. LCH: 51.4% vs. 0, p<0.001; ECD vs. RDD: 51.4% vs. 14.3%, p=0.083) were more common in ECD patients than in LCH and RDD patients. Nevertheless, the frequency of liver or spleen involvement was higher in LCH patients (32.5%) than in ECD patients (2.7%, p<0.001). No significance was found in the incidence of respiratory, dermatologic and lymph nodes involvements among three groups.
